# Molecular mechanisms of circular RNAs, transforming growth factor‐*β*, and long noncoding RNAs in hepatocellular carcinoma

**DOI:** 10.1002/cam4.2553

**Published:** 2019-09-15

**Authors:** Wenkang Shang, Gabriel Komla Adzika, Yujie Li, Qike Huang, Ningding Ding, Bianca Chinembiri, Mohammad Sajjad Ibn Rashid, Jeremiah Ong'achwa Machuki

**Affiliations:** ^1^ Department of Laboratory Medicine Eastern Hepatobiliary Surgery Hospital Second Military Medical University Shanghai China; ^2^ Physiology Department Xuzhou Medical University Xuzhou Jiangsu China; ^3^ Department of Clinical Laboratory The First People's Hospital of Kunshan Kunshan Jiangsu China; ^4^ The Third Department of Hepatic Surgery Eastern Hepatobiliary Surgery Hospital Second Military Medical University Shanghai China; ^5^ Department of Neurophysiology and Location Diagnosis Guangdong 39 Brain Hospital Guangzhou Guangdong China

**Keywords:** circular RNA, hepatocellular carcinoma, long non‐coding RNA, pathogenesis of liver cancer, TGF‐*β* signaling

## Abstract

At the heart of hepatocellular carcinoma (HCC) lies disruption of signaling pathways at the level of molecules, genes, and cells. Non‐coding RNAs (ncRNAs) have been implicated in the disease progression of HCC. For instance, dysregulated expression of circular RNAs (circRNAs) has been observed in patients with HCC. As such, these RNAs are potential therapeutic targets and diagnostic markers for HCC. Long non‐coding RNAs (lncRNAs), a type of ncRNA, have also been recognized to participate in the initiation and progression of HCC. Transforming growth factor‐beta (TGF‐*β*) is another element which is now recognized to play crucial roles in HCC. It has been implicated in many biological processes such as survival, immune surveillance, and cell proliferation. In HCC, TGF‐*β* promotes disease progression by two mechanisms: an intrinsic signaling pathway and the extrinsic pathway. Through these pathways, it modulates various microenvironment factors such as inflammatory mediators and fibroblasts. An interesting yet‐to‐be resolved concept is whether the HCC‐promoting role of TGF‐*β* pathways is limited to a subset of HCC patients or it is involved in the whole process of HCC development. This review summarizes recent advancements to highlight the roles of circRNAs, lncRNAs, and TGF‐*β* in HCC.

## Graphical abstract

Accumulating evidences implicate circular RNAs, transforming growth factor‐*β*, and long noncoding RNAs in the mechanisms of hepatocellular carcinoma. These factors carry huge potential as therapeutic targets for treatment of hepatocellular cancer.

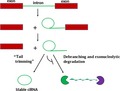

## 1 INTRODUCTION

Hepatocellular carcinoma (HCC) ranks as the third leading cause of cancer‐related deaths globally, which is associated with low survival rate.^1^, ^2^ Every year, an estimated 700 000 deaths due to HCC are recorded worldwide.^3^ Given its high recurrence and metastatic rate, HCC patients have a significantly low survival rate, which makes it a global public health burden. Currently, the main treatments for HCC are radiation, chemotherapy, surgical resection, and liver transplantation.^4^, ^5^ Some of the key risk factors for HCC include infections by hepatitis C virus (HCV) and hepatitis B virus (HBV).^6^ It has been recognized that the overall survival and life expectancy of HCC patients can be improved through early diagnosis. Late diagnosis of this disease when it has metastasized poses a great challenge for its treatment. Furthermore, effective prevention strategies and therapies for HCC are currently lacking. Consequently, it is important to explore the pathogenesis of HCC at the molecular level to help pinpoint molecular alterations that could be targeted to the diagnosis and treatment of HCC. The aim of this review was to summarize the recent findings on the features and functions of circular RNAs (circRNAs) in HCC. We focus on their influence on various processes involved in HCC development. Also, we discuss the diagnostic and therapeutic potential of circRNAs as biomarkers and targets for HCC diagnosis management.

Non‐coding RNAs (ncRNAs) refer to a class of RNA which do not code for proteins.^7^ Several types of these RNAs with diverse functional and structural features have been characterized. Based on the length of the transcript, ncRNAs are broadly categorized as long ncRNAs (lncRNAs, >200 nucleotides) and short ncRNAs (<200 nucleotides).^8^ Some key members of the short ncRNAs that are widely studied and increasingly explored are small interfering RNAs (siRNAs), piwi‐interacting RNAs (piRNAs), and microRNAs (miRNAs).^9^, ^10^ Previous studies have shown that short ncRNAs and lncRNAs are involved in many cellular processes where they modulate gene expression. For instance, lncRNAs have been found to participate in the transcriptional and translational control of gene expression by binding to RNAs, DNAs, or proteins.^11^, ^12^, ^13^ To execute these functions, miRNAs interact with specific mRNAs to induce mRNA breakdown or block their translation.^14^, ^15^ Numerous studies have reported that lncRNAs and miRNAs are frequently upregulated or downregulated in HCC, suggesting that these RNAs may be involved in HCC development and metastasis.^16^, ^17^

Recently, a type of ncRNA named circRNA has been characterized in many species.^18^, ^19^ Different from classical linear RNAs, these RNAs are formed without 3ʹ polyadenylated tails or 5 ʹ caps and have a covalently closed continuous loop structure, and hence are known to be comparatively stable relative of linear RNAs.^20^ CircRNAs are formed from pre‐mRNAs by the non‐sequential back‐splicing process, in which the upstream splice acceptor and a downstream splice donor are connected.^21^ At their discovery, circRNAs were thought to be spliced intermediates or byproducts of errant RNA splicing processes. However, the development of bioinformatics tools and high‐throughput sequencing technologies lead to the identification of several circRNAs in animals, plants, fungi, and viruses.^22^, ^23^, ^24^, ^25^, ^26^ Numerous investigations have revealed that circRNAs can act as molecular sponges for RNA‐binding proteins (RBPs) and miRNAs.^27^, ^28^, ^29^, ^30^ Additionally, circRNAs play key roles in the regulation of gene expression at the transcription level.^31^ Moreover, in many species, circRNAs are recognized to be highly conserved and their expression is influenced by developmental stage and tissue/cell types.^32^, ^33^ The characteristics of circRNAs discussed above imply that this special class of ncRNAs is likely to participate in many signaling pathways and cellular functions including pathological processes. Indeed, recent studies have implicated circRNAs in the progression of HCC. For instance, circRNAs were found to be dysregulated in patients with HCC, pointing to their involvement in tumorigenesis and metastasis of HCC.^34^, ^35^, ^36^

Therefore, these RNAs are potential therapeutic targets and diagnostic markers for HCC. This review aims to summarize the recent findings on the features and functions of circRNAs, TGF‐*β*, and lncRNAs in HCC. We focus on their influence on the various processes involved in HCC development. Their therapeutic and diagnostic potential for HCC are also explored. The ideas synthesized from this review and the molecular mechanisms explored will boost our understanding of tumorigenesis and progression of HCC which can be exploited in the design for drugs design and identification of diagnostic biomarkers for HCC.

## 2 SYNTHESIS OF CIRCRNAs

**Figure 1:**
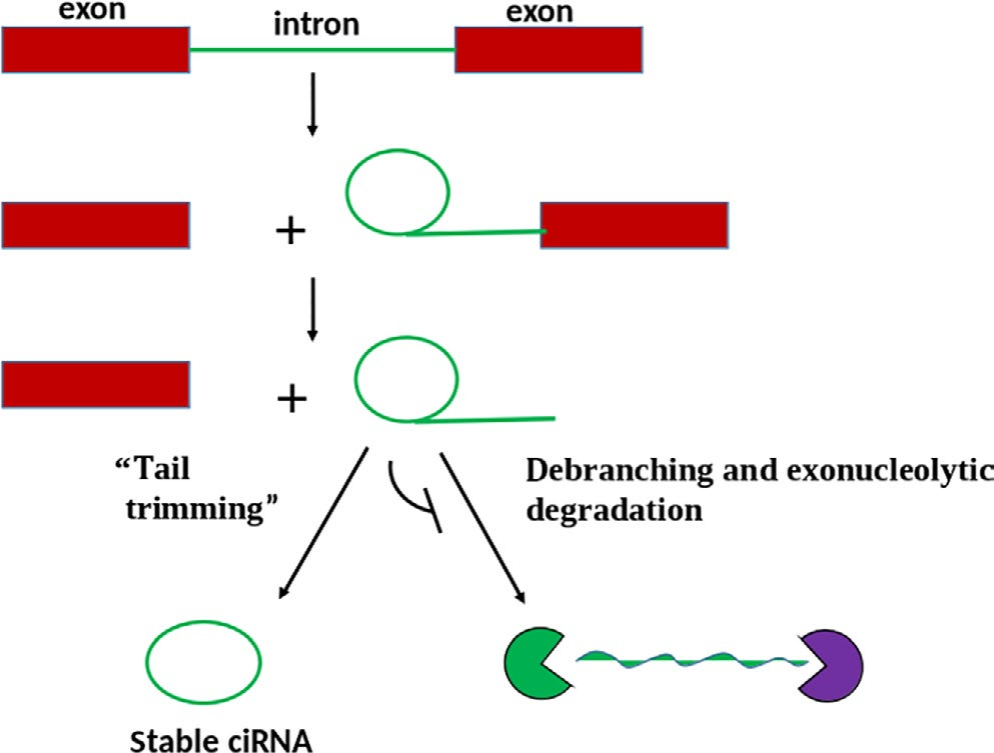
Schematic description of the synthesis of circRNAs through a spliceosome‐guided splicing. The lariat intron formed out of the splicing event escapes normal degradation and debranching, and instead the 3′ “tail” downstream from the branchpoint is cut forming a circRNA

**Figure 2:**
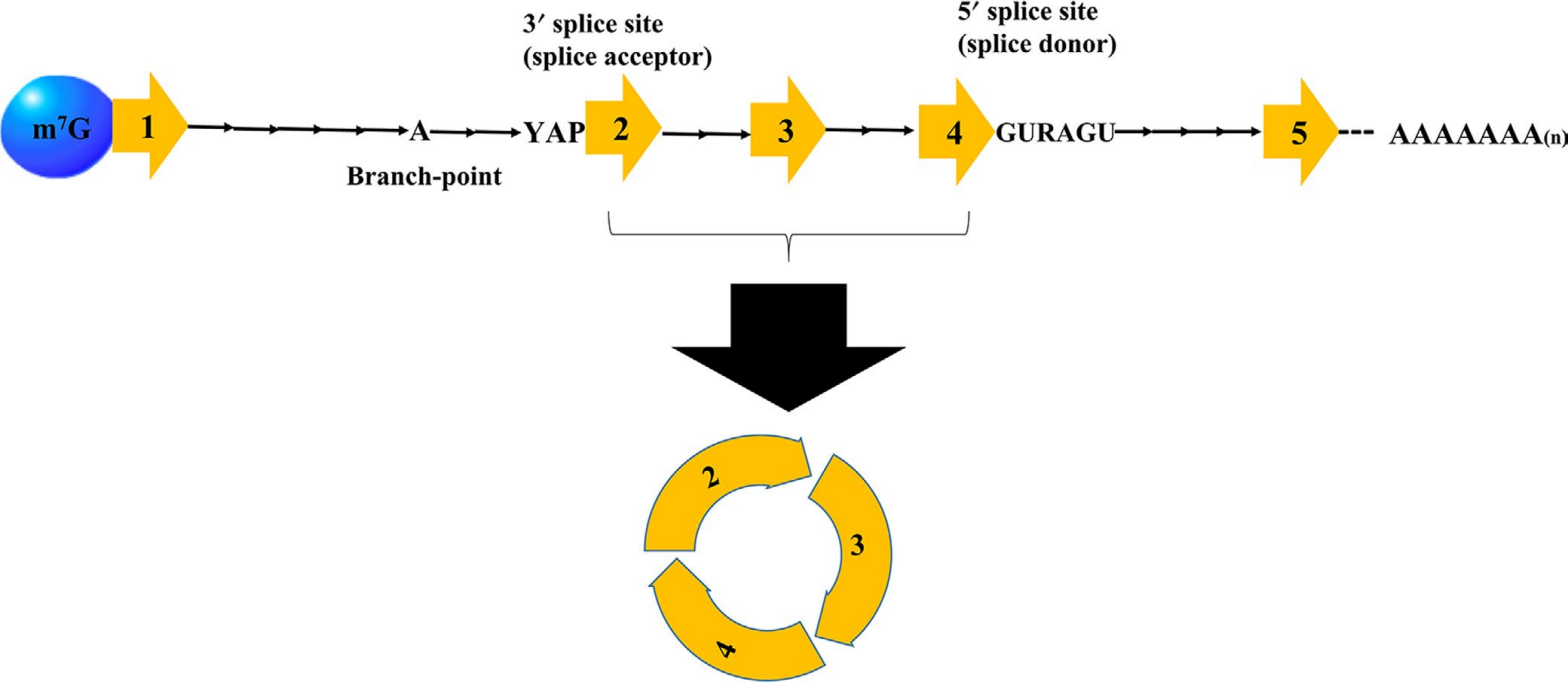
Schematic process of the formation of circRNA via back‐splicing process. A 3′ splice site (splice acceptor) of an upstream exon is joined to a 5′ splice site (splice donor) of a downstream exon through a process orchestrated by a spliceosome forming

CircRNAs are synthesized from introns, intergenic regions, exons of protein‐coding genes, antisense, or untranslated regions by back‐splicing process.^26^, ^37^, ^38^ During the back‐splicing events, exons are circularized between an upstream splice acceptor and a downstream splice donor.^39^ To date, circRNAs have been grouped into three classes: exon‐intron circRNAs (EIciRNAs), circular intronic RNAs (ciRNAs), and exonic circRNAs (ecircRNAs).^40^ The circularizing mechanisms and back‐splicing events for these circRNAs are illustrated in Figures 1 and 2. Among the circRNAs subtypes, EcircRNAs derived from exons are the majority.^41^ The formation of ecircRNA involves exon circularization of circRNA by two models namely intron pairing‐driven circularization and lariat‐driven circularization.^32^ In the latter model, distant exons within the pre‐mRNA are near each other, resulting in a lariat intermediate made of many introns and exons. Subsequently, the upstream 3ʹ splice site (splice acceptor) is joined to the downstream 5ʹ splice site (splice donor) of exons after removal of the introns. This process leads to the formation of ecircRNAs.

When introns located in the lariat are not removed by splicing, they remain in the encircled exons, forming EIciRNAs. In the intron pairing‐driven circularizing model, base‐pairing in reverse complementary sequences across exon‐flanking introns forms a circular structure. After intron pairing, pre‐mRNAs back‐splicing and exon circularization occur. Other factors that facilitate the formation of circRNA are RNA‐binding proteins (RBPs) which function as trans‐factors. Eg, Quaking (QKI), an alternative splicing factor, connects the upstream 3ʹ splice site to the 5ʹ splice site by binding to flanking introns, thereby enhancing the formation of ecircRNA.^18^ Equally, muscle blind binds to its pre‐mRNA to link two flanking introns closer to form circRNA.^21^ The RNA‐editing enzyme adenosine deaminase prevents the formation of circRNA by regulating RNA 1 (ADAR1).^42^ ADAR1 can edit the Adenosine‐to‐Inosine which mediates its role in circRNA formation. In fact, ADAR1 regulates dsRNA pairing structures and facilitates the conversion of A to I, which in turn impairs RNA pairing and inhibits back splicing for circRNA synthesis.

## 3 ONCOGENIC FUNCTIONS OF CIRCRNAs IN HCC

Several studies have implicated circRNAs in the development of HCC. A summary of studies performed to determine the aberrant expression of different types of circRNAs in HCC tissues are shown in Tables 1 and 2. Prevailing knowledge indicates that the expression of circRNAs varies with cell type/tissue. This implies that these RNAs play diverse functions in different pathological or physiological conditions.^43^, ^44^, ^45^ For instance, the oncogene CDR1as is dysregulated in many tumors such as HCC.^46^ Previously, it was found that CDR1as was highly expressed in HCC samples and it was associated with the progression of HCC and hepatic microvascular invasion (MVI).^47^ This showed that CDR1as may cause MVI in HCC patients. Indeed, the expression of CDR1as was elevated in HCC tissues relative to normal tissues.^48^ This was proven when CDR1as gene knockdown decreased HCC cells proliferation and invasion.

**Table 1:** A summary of aberrantly expressed circRNAs in HCC

CircRNA	Chromosomal localization	circBANK ID	Host gene symbol	Transcriptional change	Cellular effects	Postulated mechanism	References
hsa_circ_0001649	chr6	hsa_circSHPRH_019	SHPRH	Decreased	Apoptosis (+) Invasion (−) migration (−) proliferation (−)	Enhance expression of MMPs	[49, 50]
hsa_circ_0005075	chr1	hsa_circEIF4G3_027	EIF4G3	Increased	Invasion (−) migration (−) proliferation(−)	Sponging miRNA	[51, 52]
hsa_circ_000839	chr13	hsa_circSLAIN1_010	SLAIN1	Increased	Invasion (+) Migration (+)	regulated by miR‐200b	[53]
circZKSCAN1	chr7	hsa_circZKSCAN1_005	ZKSCAN1	Decreased	Invasion (−) Migration (−) Proliferation (−)	Modulating cancer‐related pathways	[36]
circCDK13	chr7	hsa_circCDK13_008	CDK13	Decreased	Invasion (−) Migration (−)	Regulation of JAK/STAT and PI3K/ATK signaling pathways	[54]
circARSP91	chr8	hsa_circPABPC1_023	PABPC1	Decreased	Invasion (−) Proliferation (−)	Target of ADAR1	[55]
circMTO1	chr6	hsa_circ_0007874	MTO1	Decreased	Apoptosis (+) Invasion (−) Proliferation (−)	Sponging miRNA	[56]
cSMARCA5	chr4	hsa_circSMARCA5_013	SMARCA5	Decreased	Apoptosis (+) Invasion (−) Migration (−) Proliferation (−)	Sponging miRNA	[57, 58]
hsa_circ_0005986	chr1	hsa_circPRDM2_005	PRDM2	Decreased	Proliferation (−)	Sponging miRNA	35
circC3P1	chr19	—	C3P1	Decreased	Invasion (−) Migration (−) Proliferation (−)	Sponging miRNA	[59, 60]
circSMAD2	chr18	hsa_circSMAD2_005	SMAD2	Decreased	EMT (−) Invasion (−) migration (−)	Sponging miRNA	[60, 61]
hsa_circ_100338	chr1	—	SNX27	Increased	Invasion (+) Migration (+)	Sponging miRNA	[62, 63]
circRBM23	chr14	hsa_circ_0000524	RBM23	Increased	Migration (+) Viability (+)	Sponging miRNA	[60, 64]
hsa_circ_0016788	chr1	hsa_circTRIM11_001	TRIM11	Increased	Apoptosis (−) Invasion (+) Proliferation (+)	Sponging miRNA	[65]
hsa_circ_0067934	chr3	hsa_circPRKCI_020	PRKCI	Increased	Apoptosis (+) Migration (+) Proliferation (+)	Sponging miRNA	[66]
hsa_circ_0000673	chr16	hsa_circRSL1D1_007	RSL1D1	increased	Invasion (+) Proliferation (+)	Sponging miRNA	67
circHIPK3	chr11	hsa_circ_0000284	HIPK3	Increased	Migration (+) Proliferation (+)	Sponging miRNA	34
Cul2 circRNA	chr10	hsa_circCUL2_018	CUL2	Increased	EMT (+) Invasion (+) Migration (+) Proliferation (+)	Sponging miRNA	[68]
CDR1as (ciRS‐7)	chrX	hsa_circ_0001946	CDR1	Increased	Invasion (+) Proliferation (+)	Sponging miRNA	[47, 48, 69]

**Table 2:** Function of circRNAs acts as miRNA sponge in HCC

CircRNA	circBANK ID	Host gene symbol	Postulated mechanism	Sponging miRNA	References
hsa_circ_0005075	hsa_circEIF4G3_027	EIF4G3	miRNA sponge	miR‐431	[51, 52]
circHIPK3		HIPK3	miRNA sponge	miR‐124‐3p	[34]
hsa_circ_0000673	hsa_circRSL1D1_007	RSL1D1	miRNA sponge	miR‐767‐3p	[67]
hsa_circ_0067934	hsa_circPRKCI_020	PRKCI	miRNA sponge	miR‐1324	70
hsa_circ_0016788	hsa_circTRIM11_001	TRIM11	miRNA sponge	miR‐486	66
circRBM23		RBM23	miRNA sponge	miR‐138	[65]
circMTO1		MTO1	miRNA sponge	miR‐9	[56]
hsa_circ_0001445	hsa_circSMARCA5_013	SMARCA5	miRNA sponge	miR‐17‐3p	[58]

Also, CDR1as enhances the sponging function of miR‐7. The overexpression of miR‐7 inhibited the invasion and proliferation of HCC cells in addition to decreasing the transcription of genes such as PIK3CD and cyclin E1 (CCNE1). Moreover, CDR1as enhances the proliferative capacity and invasiveness of HCC cells by acting as a sponge of miR‐7 which inhibits signaling through the PIK3CD/phospho‐p70 S6 kinase (p70S6K)/the mTOR(mammalian target of rapamycin) pathway. These findings show that CDR1as regulates the development of HCC. Furthermore, quantitative proteomics‐based approaches have revealed the presence of CDR1as‐regulated proteins in HCC cells.^69^ Results from proteomic analysis and functional verification showed that overexpression of CDR1as enhanced the cell cycle progression and proliferation of HCC cells, in part, through regulation of EGFR signaling by modulating miR‐7 overexpression. Before they become metastatic and invasive, tumor cells undergo epithelial‐mesenchymal transition (EMT), which is characterized by increased vimentin and loss of E‐cadherin.^71^, ^72^ It was reported that the EMT‐inducing transcription factor, Twist 1 increased the expression of Cul2 circRNA (circ‐10720).^68^ A positive correlation was found between circ‐10720 and tumor malignancy and poor HCC progress. Another report showed that circ‐10720 enhanced HCC cell invasion, movement, and proliferation. Mechanistically, Twist1 upregulated circ‐10720 and vimentin, by sequestering several miRNAs that target vimentin. Hence, in HCC, the Twist/circ‐10720 pathway has a positive influence on the EMT process. In contrast, silencing circ‐10720 eliminated the tumorigenic effects of Twist1 in vivo and in vitro. These results show that circ‐10720 mediates tumorigenic functions in HCC and points to its potential to treat HCC. Such findings are crucial to the exploitation of circRNA‐based therapies as alternative interventions for HCC.

Aquaporin 3 (AQP3) is an important factor in tumorigenesis and cancer progression.^73^ It is upregulated in HCC tissues, and this is thought to promote the progression and metastasis of HCC cells. In HCC cells, miR‐124‐3p expression was decreased and it was postulated that it inhibits the migration and proliferation of these cells by regulating AQP3.^34^ Elsewhere, it was stated that circHIPK3 function as a sponge of miR‐124‐3p in addition to regulating the expression of AQP3. CircHIPK3 promoted the migration and proliferation of HCC cells through the miR‐124‐3p/AQP3 axis. In vivo circHIPK3 knockdown experiments demonstrated that suppression of circHIPK3 decreased the growth of HCC cells.^67^ Another circRNA implicated in the progression of HCC is hsa_circ_0000673.^74^ This is due to the fact that it is highly expressed in HCC. Knockdown of hsa_circ_0000673 remarkably decreased the invasion and proliferation of HCC cells as well as the growth of tumor in vivo. With regard to its mechanisms, hsa_circ_0000673 is a sponge of miR‐767‐3p and hence promoted the transcription of SET, an effector of miR‐767‐3p. SET plays oncogenic roles in tumor formation since abnormal expression of SET led to enhanced cancer metastasis.^75^, ^76^ Interestingly, SET was highly expressed in HCC and was significantly linked to adverse clinical outcomes.^77^ Elsewhere, it was found that hsa_circ_0000673 enhanced HCC severity via by modulating the miR‐767‐3p/SET pathway. A previous study showed that hsa_circ_0067934 can increase invasion, migration, and proliferation of HCC cells.^66^ Mechanistically, hsa_circ_0067934 inhibits the activity of miR‐1324 thereby activating frizzled class receptor 5 (FZD5)/Wnt/*β*‐catenin signaling pathway. Taken together, these findings show that hsa_circ_0067934 promotes HCC progression and can be exploited for HCC treatment. Another investigation in which the circRNA expression profiles were screened in paired normal liver tissues and HCC tissues,^65^ revealed that 1245 circRNAs were aberrantly expressed in HCC tissues, whereby 489 were downregulated and 756 were upregulated. Among them, hsa_circ_0016788 was highly expressed in HCC tissues.

Furthermore, loss‐of‐function experiments demonstrated that silencing hsa_circ_0016788 enhanced cell apoptosis and decreased the invasion and proliferation of HCC cells. In vivo experiments revealed that hsa_circ_0016788 knockdown suppressed the spread of HCC.^65^ Furthermore, hsa_circ_0016788 acted as a sponge of miR‐486 which inversely influenced the expression of cyclin‐dependent kinase 4 (CDK4). Receiver operating characteristics analysis additionally showed that hsa_circ_0016788 exhibited a strong diagnostic value in HCC.^65^, ^67^ Taken together, the hsa_circ_0016788/miR‐486/CDK4 axis seems to regulate HCC progression, hence may be targeted to treat HCC. The transcription of CircRBM23 was elevated in HCC specimen.^64^ The high circRBM23 expression increased cell viability and elevated the migration of HCC cells. In contrast, silencing of circRBM23 suppressed the migratory and proliferative capacity of HCC cells. For instance, decreased circRBM23 levels promoted the transcription of miR‐138 and downregulated the expression of its target genes, cyclin D3 (CCND3) and vimentin. Therefore, increased expression of circRBM23 increased its oncogenic activity in HCC via the inhibition of miR‐138, a tumor‐suppressor.

In another study, four patients had their samples examined for the expression profiles of several pericancerous and HCC circRNAs.^62^ Their results revealed that 226 circRNAs were aberrantly expressed in HCC tissues, of which were decreased and 189 were remarkably increased. The high hsa_circ_100338 expression was positively correlated with significantly low survival outcome and increased metastasis in patients with HCC. Interestingly, hsa_circ_100338 acted as a miR‐141‐3p sponge in HCC. When overexpressed, it enhanced the invasiveness and migration of HCC cells. miR‐141‐3p was found to block the effects of hsa_circ_100338, thus decreasing the spread of HCC cells. These datasets imply that hsa_circ_100338 is a marker and a druggable molecule which may be exploited for the treatment or diagnosis of HCC. Additionally, miRNAs may modulate HCC progress via influencing the circRNAs transcription. In HCC tissues, it was found that the expression of miR‐200b was decreased while that of circ_000839 and Ras homologue A (RhoA) was increased in HCC.^53^ It was found that miR‐200b, circ_000839, and RhoA were negatively correlated while circ_000839 and RhoA were positively correlated. In terms of function, it was found that miR‐200b compromised the invasion and migration of HCC cells by lowering the circRNA_000839 and RhoA expression.

## 4 CIRCRNAs ARE TUMOR SUPPRESSORS IN HCC

In HCC tissues, circRNA SMAD2 (circSMAD2) expression was found to be decreased compared to adjacent normal tissues.^61^ The degree of differentiation of HCC tissues was highly influenced by circSMAD2. Overexpression of circSMAD2 inhibited EMT, invasiveness, and migration of HCC cells. miR‐629 was recognized to be influenced by circSMAD2. Furthermore, miR‐629 reversed the effect of circSMAD2 on HCC development. CircC3P1 was also downregulated in HCC.^59^ Overexpression of circC3P1 remarkably suppressed the invasion, migration, and proliferation of HCC cells. Additionally, circC3P1 also inhibited the growth of HCC and its progress in vivo. In HCC cells, the level of phosphoenolpyruvate carboxykinase 1 (PCK1) were enhanced by circC3P1 via its sponging effect on miR‐4641. Inhibiting PCK1 expression significantly blocked the effects of circC3P1 on cell invasion and proliferation of HCC. These findings show that circC3P1 plays a tumor inhibitory role by elevating PCK1 expression through regulating miR‐4641 in HCC. In HCC carcinogenesis, hsa_circ_0005986 played an inhibitory role.^35^ Moreover, the hsa_circ_0005986 expression was decreased in HCC tissues. hsa_circ_0005986 knockdown increased the levels of miR‐129‐5p which suppressed the transcription of Notch1. Another important finding is that downregulation of hsa_circ_0005986 enhanced the growth of HCC cells by activating cell cycle transition. Furthermore, some of the symptom of HCC patients such as the size of tumor, Barcelona Clinic Liver Cancer stage and MVI were to be highly associated with low expression levels of hsa_circ_0005986. Therefore, we speculate that this miRNA may be exploited as an inhibitor of HCC development and also as a diagnostic marker of HCC.

Application of the RNA‐sequencing technology to compare the transcription level of circRNAs between normal tissues and paired HCC tissues helped to identify a circRNA which was named cSMARCA5 (hsa_circ_0001445).^57^ They found that its expression was decreased in HCC tissues. Their results showed that cSMARCA5 expression was decreased in HCC tissues and this was linked to severe clinical symptoms of HCC. This points to its role as a risk factor for assessing the post‐surgery outcomes in HCC patients. cSMARCA5 overexpression decreased the migration and proliferation of HCC cells. Through its sponging roles for miR‐181b‐5p and miR‐17‐3p, cSMARCA5 promoted the transcription of a tumor suppressor, tissue inhibitor of metalloproteinase 3 (TIMP3). Based on this observation, the involvement of cSMARCA5 in the HCC tumorigenesis underscores the importance of circRNAs in HCC development. Indeed, hsa_circ_0001445 was found to be expressed in normal and HCC specimen.^58^ Its expression in HCC tissues was significantly decreased and correlated with the number of tumor foci. Gain‐of‐function experiments revealed that increased hsa_circ_0001445 expression may enhance cell apoptosis and inhibit the invasion, migration, and proliferation of HCC cells in vitro, suggesting that it plays a regulatory role in HCC disease progression. Some recent studies have investigated the expression profile of circRNAs in HCC tissues.^16^, ^56^, ^78^ Among the aberrantly expressed circRNAs, circMTO1 was found to be significantly downregulated in HCC tissues relative to its expression in normal liver tissues. The survival of HCC patients was found to be correlated with the expression of circMTO1. Indeed, overexpression of circMTO1 suppressed the invasion and proliferation of HCC cells in vitro. CircMTO1 was also found to abolish the decrease in cyclin‐dependent kinase inhibitor 1 (p21) caused by miR‐9, by sponging miR‐9. Thus, circMTO1 may partially inhibit the progress of HCC by decreasing the oncogenic activity of miR‐9. In addition, circMTO1 knockdown suppressed the growth of HCC in vivo. These observations showed that circMTO1 may be used as a prognostic marker and therapeutic avenue for treating HCC.

## 5 CIRCRNAs BLOCK THE HEPATITIS VIRUS INFECTION

Chronic hepatitis C virus and HBV infections are the key risk factors for HCC.^79^ In high incidence areas, Chronic HBV infection is the main cause of HCC which constitutes more than 50% of cases of primary liver tumors globally.^80^ HCV infection accounts for 25% of HCC cases.^81^ CircRNAs are thought to modulate the HCV or HBV infection, via influencing hepatocarcinogenesis. A previous study found that 99 circRNAs were dysregulated and were associated with chronic hepatitis B (CHB).^82^ To predict the roles of the circRNAs in CHB, CircRNA/miRNA interaction networks were constructed. Interestingly, they found that five circRNA/miRNA regulatory axes may be engaged in pathways related to HBV infection, including “MAPK signaling pathway,” “TGF‐*β* signaling pathway,” “Inflammatory mediator regulation of transient receptor potential (TRP) channels,” “T cell receptor signaling pathway” and “Hepatitis B”. miR‐122 is highly expressed in the liver.^83^ The liver‐specific miR‐122 functions in many processes such as metabolic functions, homeostasis, differentiation, and liver development.^84^ miR‐122 is also found to be involved in the regulation of genes involved in angiogenesis, EMT, hepatocarcinogenesis, such as Wnt1, serum response factor (SRF), insulin‐like growth factor‐1 receptor (IGF1R), disintegrin and metalloproteinase domain‐containing protein 10 (ADAM10) and cyclin G1. In vivo experiments have shown that suppression of miR‐122 may enhance hepatocarcinogenesis, whereas normalization of miR‐122 expression inhibited the HCC progression.^85^, ^86^, ^87^ Hence, miR‐122 acts as a tumor suppressor in the liver and miR‐122 mimics could be new strategies to treat HCC. Moreover, miR‐122 participates in the life cycle of HCV. miR‐122 stabilized HCV RNA by directly interacting with the 5**ʹ** UTR of the viral genome, which enhanced the HCV replication.^88^ Considering its function in the life cycle of HCV, miR‐122 carries huge potential as a therapeutic target for antiviral therapy. In fact, two anti‐miR‐122 drugs, RG‐101 and Miravirsen have been developed and clinically tested.^89^, ^90^

Several clinical studies have demonstrated that the inhibitors of miR‐122 play an important role in reducing viral load in HCV patients with chronic infection. Other researchers have designed circRNA sponges to absorb miR‐122.^91^ Such artificial circRNAs possess the capacity to mount inhibition on viral protein synthesis, hence leading to the suppression of HCV replication.

However, decreasing the ability of miR‐122 to suppress tumor activity using artificial circRNAs is a possibility that is worth considering. Aside from inhibiting HCV replication, circRNA sponges may also induce hepatocarcinogenesis by decreasing the miR‐122 activity. Since it plays crucial functions such as those involved in the maintenance of hepatic phenotype, lack of miR‐122 would lead to detrimental effects on the patients. To enable the clinical application of miR‐122‐suppressive therapy to treat HCV infection, the consequence of inhibiting miR‐122 on the HCC disease progression should be determined. Additionally, the cancer state and hepatic physiology should be monitored routinely during the clinical administration of anti‐miR‐122 therapy to HCV‐infected patients. Further efforts are required to design circRNA sponges targeted at miRNAs which are involved in pathogenesis and hepatitis virus replication.

Considering that circRNAs are structurally stable and are expressed in crucial cellular localizations, they may be exploited in the field of molecular medicine. Increasing evidence points to the possibility that the expression profiles of miRNA are altered during chronic HBV/HCV infection and the dysregulated miRNAs modulate the incidence of virus‐related HCC.^92^, ^93^, ^94^ Through their sponging actions on miRNAs associated with HBV/HCV infection, circRNAs are key modulators of tumorigenesis and progression of HCC. Moreover, recent reports provide evidence that circRNAs participate in the regulation of antiviral immune responses.^95^, ^96^ Hence, the effects of circRNAs on the immune system of the host may be another mechanism by which they regulate the pathogenesis of HCC due to hepatitis virus infection. Yet, the prevailing studies have not provided sufficient understanding of the relationship between HBV/HCV infection and circRNAs, which limits the full understanding of the molecular mechanisms underlying hepatocarcinogenesis caused by hepatitis virus infection. Additional research models are required to explore multiple cellular pathways of HCC targeted by circRNAs.

## 6 ROLE OF TGF‐*β* PATHWAYS IN THE REGULATION OF HCC

A study by Coulouarn et al on human tissue and mouse models revealed that TGF‐*β* signaling exhibits two types of responses, ie early and late.^97^ They reported that the early response was linked to the late response pattern and was associated with shorter survival. We speculate that the early response phenomenon indicates the development of inflammatory reactions, while the late response points to a long‐term TGF‐*β* activation in a manner equivalent to the one observed in colorectal cancer.^98^ Another mechanism by which TGF‐*β* plays an important function in HCC is the regulation of the Wnt signaling pathway. Besides, TGF‐*β* signaling seems to regulate the growth of tumor cells in some subtypes of HCC and other subtypes, it causes poor prognosis, low *α*‐fetoprotein (AFP) expression, and larger tumors.^99^

Understanding the specific HCC subgroups where these unique TGF‐*β* signaling phenomena occur is crucial in the determination of which HCC patients are likely to respond to TGF‐*β* signaling inhibition. Due to the differences among transcriptome‐based studies, there are on‐going reviews aimed at obtaining a common classification and to expand our understanding of the pathophysiologic pathways involved in hepatocellular carcinoma. The outcome of such a review of the transcriptome‐based studies may reveal that TGF‐*β* signaling is associated with EpCAM and AFP expression.^100^ Based on this concomitant expression, up to 25% of early HCC cases are likely driven by TGF‐*β* signaling. It is worth to note that transcriptome‐based assessments are based on surgical specimen following local resections and are not from advanced HCC. Therefore the interpretation of findings obtained from transcriptome‐based assessments based on liver resections should take into consideration of this concept when inhibiting the TGF‐*β* signaling in patients with advanced HCC by pharmacologic agents.

**Figure 3:**
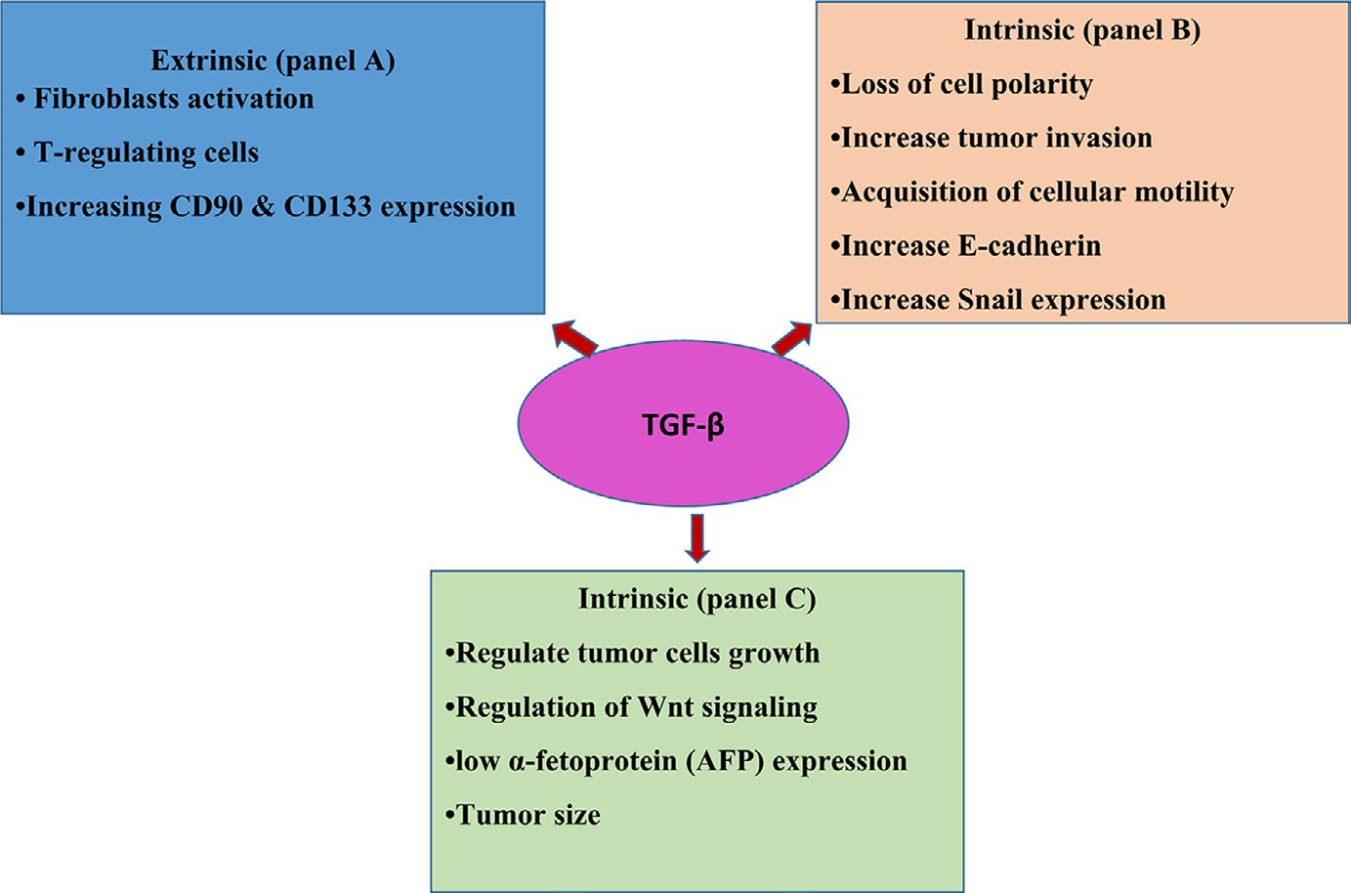
A summary of TGF‐*β* signaling in HCC

The extrinsic effect of TGF‐*β* signaling is due to tumor cell growth within the ECM‐enriched environment. The presence of tumor in the ECM is associated with connective tissue growth factor and TGF‐*β* secretion, thereby affecting the cancer‐related fibroblasts.^101^, ^102^ Moreover, TGF‐*β* signaling activates fibroblasts and is associated with T regulatory cells, for example, by activating chemokines (such as CCL22) or by the immune presentation of AFP.^103^, ^104^ Recent studies have demonstrated that TGF‐B participates in tumorigenesis.^105^, ^106^ When the liver progenitor cells are stimulated by TGF‐*β*, they are transformed into tumor‐initiating cells as evidenced by TGF‐*β*—induced changes in CD133 and CD90 expression (Figure 3).^105^ In comparison with the extrinsic effects, the intrinsic actions of TGF‐*β* signaling are largely observed in highly invasive tumor conditions (Figure 3). TGF‐*β* signaling causes increased tumor invasion, acquisition of cellular motility and loss of cell polarity.^107^, ^108^, ^109^, ^110^, ^111^, ^112^, ^113^ Alteration of the EMT process is one of the main changes in tumor cells. E‐cadherin expression is used as a biomarker of EMT. When present, TGF‐*β* causes E‐cadherin to be shed from tumor cells rendering the cells more migratory and invasive. Another component of EMT influenced TGF‐*β* is the transcription factor Snail. Snail is also induced by other factors of the tissue microenvironment such as CD44 or laminin‐5 (Ln‐5). These effects are correlated with poor prognosis.^111^, ^114^ Collectively, the data reviewed above provides evidence that TGF‐*β* signaling causes EMT and renders tumors more invasive.

## 7 LONG NONCODING RNAs SIGNALING IN HCC

Noncoding RNAs (ncRNAs) are a versatile group of RNA transcripts without protein‐coding potential.^115^ These molecules are stratified as small ncRNAs (<200 bps, eg, piRNAs, miRNAs, siRNAs) and long ncRNAs (lncRNAs) (>200 bps, eg, macroRNAs, lincRNAs).^116^ The development of modern technologies such as microarrays and high‐throughput sequencing have enabled the identification of several lncRNAs. Concerning cellular localization, lncRNAs are present in the cytoplasm or the nucleus. In these compartments, they execute multiple functions. It has been shown that many of the lncRNAs are broadly expressed in normal cells as well as in cancerous cells such as colorectal cancer, lung cancer, breast cancer, and HCC.^117^ LncRNAs participate in regulating angiogenesis, cell proliferation, metastasis, epithelial‐mesenchymal transition (EMT), autophagy, and so forth.^117^, ^118^ They can also cross‐communicate with protein molecules, RNA, and DNA, thereby regulating essential functions such as transcriptional and post‐transcriptional regulation, as well as chromatin organization. When dysregulated, they enhance the ability of tumor cells to grow and metastasis.^119^ Given that they display a cancer‐specific expression pattern, and that they can be detected in clinical specimens such as urine and blood, lncRNAs holds huge potential as biomarkers for identification of tumors. Thus, a deeper understanding of HCC‐specific lncRNAs is required to improve the management of HCC patients.

### 7.1 Upregulated lncRNAs in HCC

The roles of lncRNAs in tumors fall into oncogenic and tumor suppressive categories.^120^ A summary of studies performed to determine the aberrant expression of different types of upregulated lncRNAs in HCC tissues is shown in Table 3. Highly upregulated in liver cancer (HULC) is the first lncRNA found to be upregulated in HCC,^121^ especially in plasma of patients and cell lines,^121^, ^122^, ^123^, ^124^, ^125^ demonstrating that it may be used to identify HCC. It is also a key regulator of several biological processes such as chemoresistance, autophagy, angiogenesis, EMT, and proliferation. In addition, other reports have confirmed the association of HULC overexpression with clinical TNM‐based staging,^122^ tumor size,^126^ and overall survival as well as recurrence of HCC.^127^ HULC similarly participates in hepatitis B virus (HBV)‐induced HCC, in which hepatitis B virus X protein (HBx) has important functions.^128^ HBx elicited marked increases of cell proliferation by elevating HULC and suppressing p18, whereas HULC suppression canceled HBx‐induced cell proliferation and increased p18 expression.^124^ Collectively, these datasets indicate that HULC can be applied in the diagnosis of HCC.

**Table 3:** Upregulated lncRNAs in HCC

LncRNA	Dysregulation	Gene locus	size (bp)	Cellular functions	Clinicopathological features	Upstream regulators	Downstream targets	References
HULC	Upregulated	6p24.3	1638	Proliferation	Metastasis	HBx	p18	[129, 130, 131, 132]
						CREB	miR‐372	
HOTAIR	Upregulated	12q13.13	12 649	Chrome state	Metastasis			[129, 130, 131, 132]
					Prognosis	Suz‐Twelve	PRC2	
MALAT1	Upregulated	11q13.1	8708	Proliferation	Metastasis	TGF‐beta	Caspase‐3	[129, 130, 131, 132]
				Apoptosis	Prognosis		Caspase‐8	
				Migration			BAX	
				Invasion			BCL‐2	
				Synaptogenesis			BCL‐XL	
							PRC1	
MVIH	Upregulated	10q22‐	2146	Microvessel	Metastasis	‐	PGK1	[130, 131, 132]
		q23		growth	Prognosis			

HOTAIR (HOX transcript antisense intergenic RNA) refers to a lncRNA derived from HOXC antisense strand.^133^ The expression of HOTAIR is high in HCC tissues and cells.^134^, ^135^, ^136^, ^137^ And it has shown to affect the clinical outcomes of patients with HCC in terms of promoting recurrence risk following hepatic transplantation, causing shorter recurrence‐free survival, and predicting poor prognosis. In term of its function in HCC, HOTAIR facilitates many cellular processes such as cell chemoresistance, autophagy, glycolysis, migration, and proliferation.^138^ A study reported that HOTAIR orchestrated the suppression of miRNA‐218‐induced Bmi‐1 expression and enchained the signaling by P14 and P16, which led to increased hepatocarcinogenesis.^135^ FOXC1 increased the level of HOTAIR by inhibiting miR‐1 in HCC cells, an effect that accelerates cell proliferation. 139 Furthermore, HOTAIR may promote HBV‐ induced HCC by boosting the breakdown of ZNF198 and SUZ12.^140^ It was shown that HOTAIR triggered autophagy in HCC cells via its positive effects on ATG7 and ATG3.^141^ In conclusion, HOTAIR plays important roles in the initiation of HCC via multi‐pathway mechanisms.

MALAT1 (metastasis‐associated lung adenocarcinoma transcript 1) has been found to be expressed in human non‐small‐cell lung cancer. Its expression in HCC specimen is increased,^142^, ^143^ and it also correlates with an elevated risk of tumor recurrence following liver transplantation, which indicates that it can predict HCC recurrence.^143^ It modulates various processes such as enhancing proliferation, regulating autophagy, chemosensitivity, metastasis, and invasion in HCC tissues. Sp3 and Sp1 were found to upregulate MALAT1 while MIT (Sp1 binding inhibitor) was found to downregulate it which reveals an avenue of targeting MALAT1 using MIT.^144^ It should be noted that p53 may function as a negative regulator of MALAT1, which promotes proliferation during liver regeneration via the Wnt/*β*‐catenin pathway.^145^ The mTOR signaling pathways are considered to be a key component of the oncogenic effects of MALAT1. For instance, MALAT1 upregulates SRSF1 and activates mTOR.^146^ Other investigators reported that it can function as a circular endogenous RNA (ceRNA) for miR‐195 as well as alleviate the miR‐195‐triggered EGFR suppression leading to accelerated cell proliferation. They also stated that it activated JAK/STAT and PI3K/AKT pathways in HCC.^147^ Similar to HOTAIR and HULC, MALAT1 participates in HBx‐triggered hepatocarcinogenesis, since its expression is increased by HBx, thereby enhancing metastasis and proliferation through LTBP3 activation as well as the formation of the HBx‐MALAT1‐LTBP3 axis.^148^ To this end, we summarize that MALAT1 modulates many processes which are essential to HCC progression, and hence has the potential to be a target for HCC treatment.

MVIH (Microvascular invasion in HCC) is found on chromosome 10 and has been recognized to play a role in HCC as reported in a study by Yuan et al^149^ When highly expressed in HCC specimen, it correlated with high invasiveness and poor prognosis as well as unsatisfactory recurrence‐free survival (RFS) and overall survival (OS) outcomes.^149^ MVIH also influenced the ability of HCC tumor cells to proliferate, migrate, apoptosis, metastasize and undergo apoptosis. It also affects angiogenesis process in HCC.^149^, ^150^ Angiogenesis promoting effects of MVIH were linked to its ability to decrease in PGK1 secretion.^149^ As a sponge for miR‐199, MVIH orchestrated the suppression of miR‐199 leading to decreased apoptosis and cell proliferation in HCC cells.^150^ A recent study showed that MVIH controls migration and proliferation of HCC cells by modulating ARID1A‐induced effects on CDKN1A.^151^ These findings reveal that MVIH possesses the oncogenic potential and it can be exploited to diagnose and predict tumor recurrence in HCC patients.

### 7.2 Downregulated lncRNAs in HCC

Prior studies have supplied compelling evidence that some ncRNAs such as LET, Dreh, MEG3, and H19, are important players in tumor suppression. Accordingly, their expression level in HCC is decreased. The studies that were designed to investigate the effect of downregulated lncRNAs in HCC are summarized in Table 4. In HCC, H19 modulates many aspects of disease progression. Alteration in its expression was found to be associated with disease‐free survival and outcomes in patients.^152^, ^153^, ^154^ In terms of function, it was reported that H19 was involved in regulating chemoresistance, metastasis, EMT, invasion, migration, and migration of HCC cells.^149^, ^153^, ^155^, ^156^ Moreover, when expressed, H19 promoted the in vivo tumor growth, whereas its inhibition produced opposite effects.^156^ Further investigation demonstrated that H19 caused poor prognosis and disease‐free survival when it was elevated in HCC patients. This indicates that H19 can be used to assess the prognosis of HCC.^154^ But, there are reports where H19 expression is decreased in HCC,^153^, ^154^ and reflect poor prognostic outcomes.^153^ Other studies implicated H19 in the activation of miR‐200 and suppression of EMT process and tumor metastasis.^153^ In a study where miR‐675 was used to inhibit H19, results showed that this enhanced metastasis of HCC through the AKT/GSK‐3beta/Cdc25A pathway.^149^ To this end, it can be concluded that H19 mediates a suppressive effect on tumors and may be an oncogene in HCC.

**Table 4:** Downregulated lncRNAs in HCC

LncRNA	Dysregulation	Gene locus	size (bp)	Cellular functions	Clinicopathological features	Upstream regulators	Downstream targets	References
H19	Downregulated	11p15.5	2660	Metastasis	Prognosis		miR‐200	[129, 131, 157]
				Proliferation				
MEG3	Downregulated	14q32.3	34 919	Proliferation	Prognosis	cAMP	p53	[129, 130, 131, 132]
Dreh	Downregulated	17q	1402	Cytoskeleton	Prognosis	HBx protein	Vimentin	[130, 131, 158]
				structure				
LET	Downregulated	15q24.1	2606	epigenetic regulation	Prognosis		miR‐138	[129, 131, 159]

Maternally expressed 3 (MEG3) encodes lncRNA with a maternal inheritance pattern lncRNA.^160^ This lncRNA regulate HCC cells apoptosis and proliferation.^161^, ^162^, ^163^, ^164^ Its transcription is low in human HCC tumors^161^, ^162^, ^165^ and was correlated with low OS levels, making it a potential predictor of HCC prognosis.^162^ Among the mechanisms by which MEG3 inhibits tumor growth and development include its ability to activate p53 through a process involving promoting the stability and expression of genes.^161^, ^163^ Previously, MEG3 was incorporated into HCC cells by using a new delivery system, which suppressed tumor growth through p53. This finding showed that MEG3 may have tumor suppressive function in HCC.^163^ Also, MEG3 was identified to be a molecular sponge for miR‐664 inhibiting cell proliferation via miR‐664‐dependent ADH4 regulation.^166^ In summary, these findings implicate MEG3 as an anti‐tumor agent, which can be exploited in the diagnosis as well as in the treatment of HCC.

A study found that HBx or Dreh was downregulated using a lncRNA microarray assay on WT and HBx‐transgenic mice model.^167^ Its expression in tumor tissue specimen from HBV‐related HCC patients.^167^, ^168^ In this case, it was found to be associated with poor survival.^167^ Elsewhere, Dreh was recognized to participate in metastasis and proliferation of HBV‐related HCC. Furthermore, a prior investigation reported a negative correlation between HBx or HBs and Dreh expression.^168^

HBx‐induced downregulation of Dreh relied on vimentin downregulation, and the consequence of these effects was suppressed HCC cell migration and growth,^167^, ^168^ this reveals that Dreh acts as a tumor suppressor in HBV‐related HCC. “Low expression in a tumor” (LET) refers to a lowly expressed molecule in HCC tumor tissues.^169^ LET influenced the metastatic and invasiveness of HCC cells. LET expression is inhibited by HDAC3,^169^ and this elevated stability of NF90, thereby enhancing hypoxia‐induced invasion.^169^ This finding was confirmed in HCC clinical specimen with upregulation of NF90, downregulation of LET, and abnormal histone acetylation. Collectively, the datasets presented above reveal the anti‐tumor roles of LET in hypoxic conditions.

### 7.3 The therapeutic and diagnostic potential of lncRNAs in HCC

So far, lncRNAs have been shown to act as either oncogenes or tumor suppressors in the initiation of hepatocarcinogenesis. Intriguingly, aberrant lncRNAs expression correlates with various aspects of cancer such as tumor‐node‐metastasis(TNM) stage, RFS, disease‐free survival (DFS), OS, metastasis, and proliferation. By multivariate analysis of various factors associated with HCC, it was found that lncRNAs can independently predict outcomes and recurrence of HCC. Given the recent advancements in the tools of diagnosing cancers such as RNA immunoprecipitation, microarrays, qRT‐PCR, and sequencing technology, it is now possible to detect lncRNAs in different types of body fluids, which is likely to boost their application as prognostic markers of HCC. For instance, it was demonstrated that HULC was markedly increased in tumor tissues and serum of HCC patients; hence, it holds huge promise in the diagnosis of HCC.^125^, ^126^ Aside from plasma lncRNA, exosomal lncRNA may be used as biomarkers. A study reported that HEIH, an oncogenic lncRNA, was highly expressed in exosomes and sera of subjects with HCV‐related HCC. Further studies are required to identify other lncRNAs with the potential to be biomarkers of HCC.

Given that several lncRNAs together with associated signaling molecules are dysregulated in HCC, strategies that restore their normal cellular levels are likely to provide newer cancer treatments, which are less susceptible to chemoresistance. Indeed, various drug companies have directed many resources to exploit the potential of lncRNAs as drug targets.^170^, ^171^ The expression of lncRNAs can be manipulated by specific siRNAs or antisense oligonucleotides or exogenous overexpression.^172^, ^173^ Previously, a study demonstrated that introduction of tumor suppressor MEG3 into HCC tumor via a novel delivery system promoted apoptosis,^163^ an effect that confirms the pharmacological value of lncRNA‐based therapy as an option with few adverse effects. Hence, we anticipate that further advanced studies exploiting modern research tools will expose deeper mechanisms of lncRNA action and add to the development of lncRNA‐based diagnostic and therapeutic agents for HCC management.

## 8 CONCLUSION AND FUTURE PERSPECTIVES

The pathogenesis of hepatocellular carcinoma is characterized by multiple causes. The several non‐coding RNAs are deregulated at various stages of HCC. CircRNAs and lncRNAs exhibit diverse associations with proteins, RNAs, and DNAs and thereby playing crucial roles in post‐transcriptional, transcriptional and chromatin organization regulation of HCC cells. CircRNAs and lncRNAs have shown a high potential to be used as markers of HCC or diagnosis. Several therapies such as inhibitors of the TGF‐*β* signaling have shown high efficacy in preventing HCC progression via their modulatory roles on the EMT process. In fact, an inhibitor of TGF‐*β*, LY2157299, has been clinically investigated in HCC and found to have improved outcomes. When aberrantly expressed, lncRNAs renders cells more likely to undergo tumorigenesis, metastasis and growth diseases and are responsible for a defective immunosurveillance, leading to HCC emergence. This review also reveals that circRNAs show HCC tissue‐specificity. The functions and level of circRNAs may be correlated with metastasis, TNM stage and tumor size in patients with HCC, hence may serve as indicators of stage phenotypes of HCC progress. Thus, circRNAs can be exploited to improve clinical HCC diagnosis as they are effective in distinguishing cancerous from normal tissues. However, for this to be achieved, large‐scale clinical trials should be carried out to evaluate their clinical utility. Much of the circRNAs measurements in HCC have been performed using tissues from patients. Further studies should develop isolation protocol based on non‐invasive clinical samples such as urine, saliva, blood etc

## Data Availability

All data pertaining to this manuscript is provided in the manuscript and is available on request from the authors
